# The influence of home and environmental characteristics on 5–18 years old children's health during the COVID-19 pandemic: A cross-sectional study in Iran

**DOI:** 10.3389/fpubh.2023.1134411

**Published:** 2023-03-30

**Authors:** Majid Golzarpour, Paula Santana, Homeira Sajjadi, Gholamreza Ghaed Amini Harouni, Claudia Costa, Arash Ziapour, Seyed Amar Azizi, Mehdi Akbari, Sima Afrashteh

**Affiliations:** ^1^Student Research Committee, University of Social Welfare and Rehabilitation Sciences, Tehran, Iran; ^2^Social Welfare and Health, University of Social Welfare and Rehabilitation Sciences, Tehran, Iran; ^3^Centre of Studies in Geography and Spatial Planning, Department of Geography and Tourism, Humanities Faculty, University of Coimbra, Coimbra, Portugal; ^4^Social Determinants of Health Research Center, University of Social Welfare and Rehabilitation Sciences, Tehran, Iran; ^5^Social Welfare Management Research Center, University of Social Welfare and Rehabilitation Sciences, Tehran, Iran; ^6^Cardiovascular Research Center, Health Institute, Kermanshah University of Medical Sciences, Kermanshah, Iran; ^7^Urban Planning, University of Tehran, Tehran, Iran; ^8^Clinical Research Development Center, The Persian Gulf Martyrs Hospital, Bushehr University of Medical Sciences, Bushehr, Iran

**Keywords:** home environment, environmental characteristics, children's health, COVID-19, Iran

## Abstract

**Background:**

The coronavirus disease (COVID-19) pandemic has dramatically changed the health and wellbeing of children. Therefore, this study aimed to investigate the relationship between the home environment and the environmental characteristics on 5–18 years old children health in Iran.

**Method:**

An online survey was conducted among parents of children aged 5 to 18 living in large cities in Iran in 2021. The statistical population of this cross-sectional study was 500 people. In this survey, questionnaires on the quality of the home environment, exterior and interior landscapes of homes, and the Child Health Questionnaire (CHQ) were used to investigate the relationship between the home environment and environmental characteristics on 5–18 years old children health during the COVID-19 pandemic. The *t*-test and analysis of variance were used in SPSS 24, and the structural equation modeling (SEM) was utilized in AMOS 24 for analyzing the data.

**Results:**

The average age of respondents was 37.13 ± 7.20, and that of children was 11.57 ± 3.47. 73.02% of the families were covered by insurance, and 74.08% of them lived in the metropolis. In addition, 65.04% of the families complied with the restrictions of the quarantine period. A share of 31% of the families live in villas, and 55% paid more attention to cleaning their homes during the COVID-19 pandemic than before. A positive and significant statistical relationship (β = 0.414, *p* < 0.001) was observed between the residence environment and child health. Thus, explained 17.5% of variations in child health.

**Conclusion:**

The results showed that the children who lived in homes with an exterior landscape in nature had better health. In addition, the 5–18 years old children whose home landscape was a garden, compared to the other two groups (yard, balcony), had better health. Gardens are a potential source of health and not necessarily replaced by other natural environments, thus providing them along with green space is one of the crucial issues that should be considered.

## Introduction

The COVID-19 pandemic has dramatically changed the health and wellbeing of children, especially those who are isolated indoors due to social distancing measures (1–3). In this regard, public health measures necessary to deal with the COVID-19 pandemic have led to significant changes in the physical and social environments in which children grow up (4, 5).

Harm to children affected by COVID-19 can manifest itself in multiple and often hidden ways, and by focusing exclusively on the health effects of the infection, most of the impact of this disease on children's lives has been ignored. Although children are more resistant to the disease, the epidemic has affected various aspects of their daily life and may also affect their health and development (4, 6). In this regard, in addition to the economic effects caused by the tense labor market, the health measures required to deal with the COVID-19 disease have led to significant changes in the environments in which children grow and develop (4, 7–9).

The family, school, or general environment in which a child lives and interacts affects her or his growth and development. Children in the early formative years pick up things from their environment, acquire habits and behaviors, socialize, and function. Consequently, studying the main environmental factors affecting the growth and development of a child is necessary. These factors include the social, emotional, economic, and physical environment (10, 11). The physical environment is the space and location in which the child grows and which affects her or his health, learning, and behavior. In addition, research shows that the effects of the physical environment, such as housing, exposure to pollution, and neighborhood quality, all affect the psychosocial aspect of the child (12, 13).

As our understanding of methods of exposure to the virus and associated health outcomes in children increases, it is crucial to consider disease-related changes in broader settings (exterior landscapes of homes) such as nature, gardens, yards, balconies, and gardens as a potential source of health and not necessarily replaceable with other natural environments that can affect children's development now and in the future. It is believed that staying at home and social distancing measures have had adverse effects on the physical-social environment of children, especially those who have already been exposed to environmental injustices due to socioeconomic and health conditions (1, 14–18).

Of all the aspects of the life of COVID-19 infected children, the discussion of physical environments, particularly home environment conditions and their exterior landscapes, has received less attention to date. Although stay-at-home guidelines prevented the spread of COVID-19, these mandates can lead to greater exposure to indoor pollutants and exacerbate pre-existing conditions for many children. Studies have shown that changes in the environmental conditions of children can be directly or indirectly related to epidemic disease (4). Air pollution is a serious community health risk (14). Evidence also suggests that air pollution is a significant risk factor for disease burden (15). Air pollution substantially contributes to premature mortality and disease burden globally, with a higher impact in low-income and middle-income countries than in high-income countries (19, 20).

Changes in children's physical environment, resulting from disruptions in services, welfare, and infrastructure, can exacerbate the social and economic inequalities they are already exposed to by the pandemic (21). To understand the consequences related to 5–18 years old children's health from exposure to the virus, it is crucial to consider how changes in home environment conditions and their exterior landscapes can affect children's development (4). Studies have shown that the physical, chemical, and biological aspects of home environment conditions and their exterior landscapes can affect many aspects of 5–18 years old children's health and development (22).

Furthermore, serious public health emergencies, e.g., pandemics, take a toll on physical and mental health. Children are especially vulnerable because of their limited understanding of the event. They may not be able to communicate their feelings like adults. Closure of schools and separation from friends can cause stress and anxiety in children. Exposure to mass media coverage of crisis events and unverified information circulating on social media may cause mental distress (23–25). A child's response to a crisis depends upon his or her prior exposure to emergencies, physical and mental health, socio-economic circumstances of the family, and cultural background (23, 25). Different studies have shown that crisis events negatively impact the psychological wellbeing of children. Anxiety, depression, disturbances in sleep and appetite, and impairment in social interactions are the most common presentations. Recent research in China screened children and adolescents for behavioral and emotional distress due to the COVID-19 pandemic. Clinginess, distraction, irritability, and fear that family members could contract the deadly disease were the most common behavioral problems identified (24).

Studies have shown that home environment conditions and exterior landscapes during the epidemic affect 5–18 years old children's health (1). Despite acknowledging the importance of home environment conditions and exterior landscapes for health, there are relatively few studies on their effects on health and even fewer on 5–18 years old children's health (26). Iran is a developing country where, like other countries in the world, the spread of COVID-19 has caused changes in the living conditions of children, and their lives have faced many dangers (27–29). Therefore, this study aimed to investigate the relationship between the home environment and environmental characteristics on 5–18 years old children health during the COVID-19 pandemic in Iran.

## Materials and methods

In this cross-sectional study, the statistical population was parents with children aged 5–18 living in large cities in 2021. Samples were selected from the residents of Iran's largest cities: Mashhad located in the Northeast, Kermanshah in the West, Zahedan in the East, Tabriz in the Northwest, and Chaharmahal and Bakhtiari in the center.

In total, 500 families with children and adolescents aged 5–18 years were selected to participate in the survey (*n* = 500). The inclusion criteria were having lived in one of the studied cities for at least 1 year, having a child between 5 and 18 years old, and having sufficient literacy to complete the questionnaires. The exclusion criteria were having a mentally disabled child or with a severe physical illness, unwillingness to participate in the study, and incomplete filling out of the questionnaire.

In this study, we used a web application (web app) to invite participants to complete an online questionnaire and anonymously collect data. We distributed the link to the questionnaire *via* email and social networking platforms such as WhatsApp, Telegram, and Instagram. We also asked respondents to circulate the link among their professional and personal networks. We put a contact number in the questionnaire link to answer the respondents' doubts about the research questions. Due to the design of the questionnaire and its distribution strategy, the response rate cannot be determined because it is impossible to estimate how many people were reached by social media, media outlets, or email.

### Study instruments

The demographic information used were gender, age, education, type of housing (apartment, villa, rental house, living with the spouse's family, institutional housing, national housing plan), features of quarantine of all family members, living in a metropolis, compliance with house cleaning during COVID-19, and insurance coverage.

### Quality of the home environment

The COVID-19 checklist for families and communities was used to measure the effects of the home environment. This checklist is one of the most reliable checklists and has been translated in 13 languages (30). We used 12 questions that evaluate conditions of the living environment, family, and community. The Principal Component Analysis (PCA) technique was used to investigate and identify the number of factors and items related to each factor. According to the results, it was found that the size and KMO = 0.779 of the sample are sufficient. Based on the results of the PCA technique, three factors were identified. These three factors explained a total of 66.70% of the total variance.

### Exterior and interior landscapes of homes

The exterior and interior landscapes of the home were measured through the COVID-19 checklist for families and communities (30), namely: 1. Which of the following are the exterior landscape of your home? A. nature B. square/street C. garbage places, 2. Your home environment includes which of the following? A. garden B. yard C. terrace.

### Child health

The CHQ is an internationally recognized health-related quality-of-life measurement instrument for 5–18 years old children. This scale was designed by Landgraf (31). It is one of the most widely used scales related to health and health-related quality of life for children (32). A 28-question form was used to assess 5–18 years old children's health status in this study. Golzarpour et al. (33) investigated its construct validity in Iran, and it was reduced to 22 questions. The questions were graded based on the Likert scale (some questions range from 1 to 4, and others from 1 to 5). In the present study, Cronbach's alpha coefficients for the components of mental health, child satisfaction, child movement, child performance, parental concern, parental restrictions, and child's general health were 0.993, 0.900, 0.891, 0.908, 0.897, 0.888, and 0.927, respectively.

### Data analysis

The *t*-test and analysis of variance were performed in SPSS24 for the data analysis. Furthermore, SEM was performed in AMOS24 to examine the research model.

### Ethics approval and consent to participate

The principal investigators conducted this study in accordance with the Helsinki Declaration and followed the ethical standards for scientific research procedures. The Ethics approved by Committee at the University of Social Welfare and Rehabilitation Sciences in Tehran (IR.USWR.REC.1400.058). All participants were informed about the study, and only those providing written informed consent were enrolled.

## Results

### Examining demographic characteristics

The sample population comprised of 195 (39%) men and 305 (61%) women (Table 1). The average age of the respondents was 37.13 ± 7.20, and 58.08% of the participants had a university degree. The average age of the children was 11.57 ± 3.47. The majority of the children of the participants in the study had primary education (58.1%). It was found that 73.02% of the families were covered by insurance, and 74.08% of them lived in the metropolis. It was found that 65.04% of the families have complied with the restrictions. Participants residing in villas were 31%. In addition, 55% of the families paid more attention to home cleanliness during the COVID-19 pandemic than before (Table 1).

**Table 1:** Sociodemographic information of study participants.

**Variable**	**Category**	***N* (%)**	**Child's total health score**	***P*-value**
			**Mean** ±**SD**	
Parents' gender	Male	195 (39%)	64.02 ± 25.68	0.857^*^
	Female	305 (61%)	62.42 ± 23.44	
Age	Parents	500 (100%)	37.13 ± 7.20	0.550^**^
Parents' education	Primary education	50 (10%)	57.10 ± 25.73	0.025^***^
	Middle school education	45 (9%)	59.84 ± 24.62	
	High school education degree	111 (22.02)	62.71 ± 22.86	
	Academic degree	294 (58.08)	66.39 ± 24.47	
Child's gender	Male	254 (50.08)	62.41 ± 23.78	0.083^*^
	Female	246 (49.02)	66.17 ± 24.75	
Age	Child	500 (100)	11.57 ± 3.47	0.550^**^
Child's education	Primary school	283 (56.06)	62.52 ± 24.28	0.157^***^
	Middle school	123 (24.06)	66.46 ± 23.85	
	High school education degree	94 (18.08)	65.53 ± 25.37	
Insurance	Yes	366 (73.02)	62.86 ± 24.43	0.035^*^
	No	134 (26.08)	60.47 ± 23.66	
Living in a metropolis	Yes	374 (74.08)	65.62 ± 23.78	0.031^*^
	No	126 (25.02)	59.41 ± 25.69	
Quarantine of all family members	Yes	327 (65.04)	66.11 ± 25.26	0.019^*^
	No	173 (34.06)	60.77 ± 25.05	
Type of housing location	Apartment	107 (21.04)	59.83 ± 22.63	0.041^***^
	Villa	155 (31%)	68.15 ± 23.99	
	Rental house	113 (22.06%)	65.31 ± 25.88	
	Living with your own family, or the spouse's family	83 (16.06)	61.46 ± 24.58	
	Institutional housing	13 (2.06)	51.92 ± 26.75	
National housing plan (Mehr Housing Project)	29 (5.08)	65.72 ± 22.22
Cleaning the living space	We do less cleaning at home than when the coronavirus spread in the country.	62 (12.04)	58.48 ± 23.14	0.020^***^
	We clean the house like before the spread of coronavirus in the country.	163 (32.06)	62.12 ± 24.06	
	We do more cleaning at home than before the spread of coronavirus in the country.	275 (55%)	66.83 ± 24.45	

^+^Independent *t*-test.

^*^p < 0.05; ^**^p < 0.02; ^***^p < 0.01.

^#^ANOVA.

According to the results of the fitted model (CMIN = 3.97, GFI = 0.916, CFI = 0.983, RMSEA = 0.077), there was a positive and significant statistical relationship between the residence environment and child health (β = 0.414, *p* < 0.001). Thus, the residence variable explained 17.5% of variations in child health (Table 2).

**Table 2:** Results of examining the model of the effects of the home environment on child health.

**Consequence**		**Determinant**	**β**	**S.E**.	**C.R**.	***P***	***R* square**
Child health	<–	Home environment	0.414	0.443	3.939	0.001	0.175
En. Fa	<–	Home environment	0.436	0.133	3.935	0.001	–
He. Re.Fa	<–	Home environment	0.585	1.476	2.435	0.013	–
Hea.Fa	<–	Home environment	0.171	0.114	2.436	0.015	–
M.H	<–	Child.Health	0.957	0.032	48.178	0.001	–
S.Sat	<–	Child.Health	0.946	0.022	45.501	0.001	–
Mov.Sat	<–	Child.Health	0.917	0.018	39.841	0.001	–
Perf	<–	Child.Health	0.961	0.023	49.080	0.001	–
Uneasy	<–	Child.Health	0.934	0.016	42.949	0.001	–
Par.Lim	<–	Child.Health	0.934	0.016	42.936	0.001	–
G.H	<–	Child.Health	0.945	0.022	45.501	0.001	–

The results showed that homes with the exterior landscape of nature, compared to the other two groups (square/street, garbage places), were statistically significant and had better health in each dimension of child health (Table 3). The findings showed that children at homes with the exterior landscape of nature, compared to the other two groups (square/street, garbage places), had better health status (Table 3).

**Table 3:** Results of the effects of exterior landscapes of the home on 5–18 years old children health.

**Total score**	**Exterior landscape**			***F***	***P*-value**
	**Nature**	**Square/street**	**Garbage places**		
	**Mean** ±**SD**	**Mean** ±**SD**	**Mean** ±**SD**		
Child's mental health	16.31 ± 5.82	14.90 ± 5.71	11.50 ± 3.92	8.94	<0.001
Child's self-satisfaction	9.74 ± 3.73	9.02 ± 3.71	6.58 ± 2.87	8.35	<0.001
Child's movement status	7.68 ± 2.87	7.03 ± 2.75	5.75 ± 2	6.57	<0.002
Child's performance	11.52 ± 4.23	10.68 ± 4.21	8.62 ± 3.29	5.94	<0.003
Parental concern	6.62 ± 2.55	5.98 ± 2.58	4.41 ± 2.16	9.17	<0.001
Parental limitations	6.59 ± 2.54	5.98 ± 2.56	4.95 ± 2.45	6	<0.003
Child's general health	10.17 ± 2.76	9.15 ± 3.75	7.20 ± 2.93	8.71	<0.001

Some variables could not enter the regression model due to the type of measurement scale (nominal). Therefore, their relationship with child health was investigated separately (Tables 3, 4).

**Table 4:** Results of the effects of the interior landscapes of the home environment on 5–18 years old children's health.

**Total score**	**Location view**			***F***	***P*-value**
	**Garden**	**Yard**	**Terrace**		
	**Mean** ±**SD**	**Mean** ±**SD**	**Mean** ±**SD**		
Child's mental health	17.25 ± 6.36	15.43 ± 5.57	13.96 ± 5.21	13.49	<0.001
Child's self-satisfaction	10.46 ± 4.13	9.22 ± 3.74	8.37 ± 3.27	12.84	<0.001
Child's movement status	8.00 ± 3.02	7.38 ± 2.85	6.61 ± 2.49	10.48	<0.001
Child's performance	12.24 ± 4.68	10.89 ± 4.08	10.10 ± 3.84	10.41	<0.001
Parental concern	6.94 ± 2.71	6.15 ± 2.63	5.67 ± 2.39	9.71	<0.001
Parental limitations	7.02 ± 2.80	6.27 ± 2.53	5.57 ± 2.32	13.26	<0.001
General health of the child	10.68 ± 4.15	9.50 ± 3.74	8.65 ± 3.38	11.79	<0.001

The results showed that the homes with a garden, compared to the other two groups (yard, terrace), were statistically significant and had better health in each of the dimensions of child health (Table 4).

### Examining the research model

A regression model with a structural equation modeling method was used to investigate the relationship between the residential environment and child health.

According to the results of the fitted model (CMIN = 3.97, GFI = 0.916, CFI = 0.983, RMSEA = 0.077), there was a positive and significant statistical relationship between the residence environment and child health (β = 0.414, *p* < 0.001). Thus, the residence variable explained 17.5% of variations in child health (Table 2).

## Discussion

Children have experienced many changes in their physical and social environments during the outbreak of COVID-19 (34). Just as environmental exposure to stressful social and economic dimensions interacts with our condition, its effects on children's health can be amplified and repeated for a long time, perhaps over generations. In this regard, according to the authors' knowledge at the time of writing this paper, no study was found that investigated the home environment conditions and environmental landscapes on child health in Iran. Therefore, this study is the first research done in this field in Iran.

**Figure 1:**
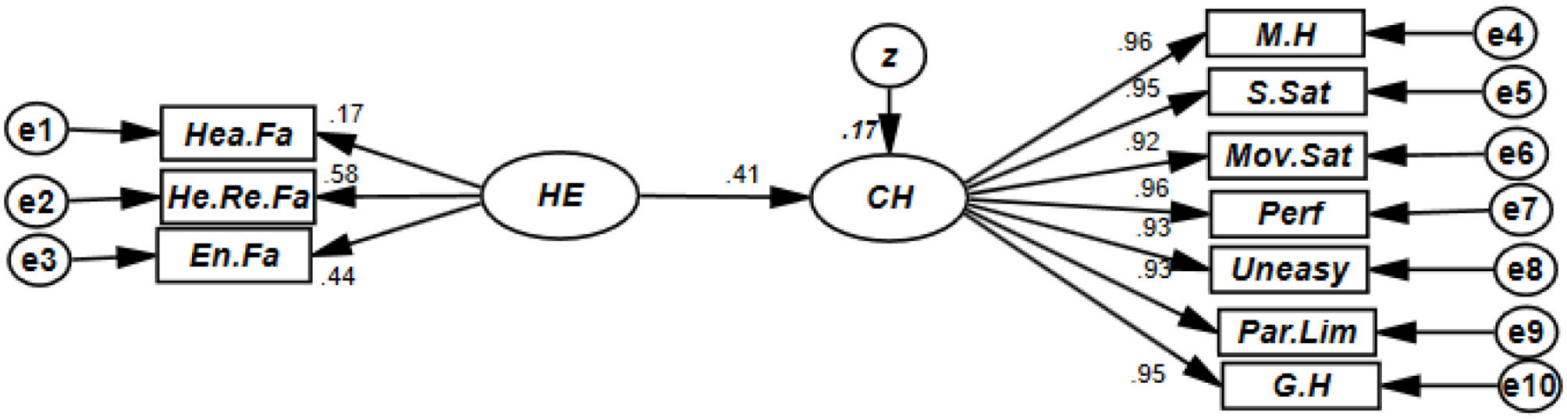
Theoretical model of research. Model guide: Home enviorment: He.Fa, heating factors; He.Re.Fa, health-residential factors; En.Fa, environmental factors. Child health: M.H, child's mental health; S.Sat, child's self-satisfaction; Mov.St, child's movement status; Perf, child's performance; uneasy, parental concern; Par.Lim, parental limitation; G.H, child's general health.

The results of our study showed a statistically significant relationship between the residential environment and children's health (Figure 1). Other studies also showed that the home environment status affected children's growth (35). Inadequate living conditions can expose children to several important infectious childhood diseases and poor physical development outcomes (36). Another study showed that during the quarantine when there was a smoker in a household, his or her family might have suffered from second-hand smoke more frequently. It was especially severe for children more sensitive to air pollution. Playing at home or cleaning the floor could also increase indoor air pollution. Human activities were more frequent during the quarantine since families stayed together at home almost the whole day. COVID-19 quarantine environmental damage because air pollutants might be aggravated. From our viewpoint, the quarantine might fail to save people's lives by improving ambient air quality because when indoor air pollution is taken into consideration, it may have a negative effect (37). Therefore, in this regard, we suggest that future studies investigate how continuous changes in the residential environment can improve children's health.

The results of this research show that the children of people whose interior landscape was nature had better health (Table 3). Other studies have also shown that nature around the home may play a key role in reducing the adverse mental health consequences of the COVID-19 pandemic and the measures taken to deal with it. Nature has been associated with increased self-esteem, life satisfaction, mental happiness, and reduced levels of depression and anxiety (38, 39). Contact with nature helps people cope with the effects of depression and anxiety caused by the COVID-19 disease, especially for those under strict quarantine (38). Indeed, nature can be used as a “nature-based solution” to improve public health during pandemics such as COVID-19 (40). This issue is especially crucial during the outbreak of COVID-19 when people experience higher stress levels and are confined to their homes in isolation (39). The use of green space probably encourages children to do physical exercises, which in turn helps to improve their mental health (37). In addition, outdoor nature provides opportunities for children to interact with other members of local communities (for example, friends), which may have alleviated their isolation and loneliness and increased their sense of wellbeing (41). It may be less effective than usual due to the social distancing practices imposed during the pandemic but interactions such as seeing other people or sending messages to others from an acceptable distance help to decrease adverse social psychological effects of epidemics (39). These results can help decision-makers develop potential quarantine measures in the future to reduce adverse impacts by helping people to be more resilient and maintain better mental health using the benefits that ecosystem services offer (38).

The results show that the children's health of people whose home environment had a garden, compared to the other two groups (yard, terrace), had better health. Other studies have shown that people who use green spaces such as gardens have better health and wellbeing and more physical activity. These findings suggest that home gardens are a potential source of health and do not necessarily replace other natural environments (42). These results have important implications for the planning and development of urban areas and provide evidence that there is a need for private green spaces and open spaces alongside publicly accessible green spaces. On the other hand, the home garden, a type of small green space, can provide ecosystem services with ecological functions in reducing mental stress during the isolation period of the COVID-19 pandemic through physical activities. A home garden, a small green landscape with biodiversity content, allows people to get close to nature to create a confortable and natural feeling during the COVID-19 pandemic. Furthermore, such an eco-friendly home garden approach that favors urban biodiversity can deal with the challenges of maintaining environmental and mental health in the recovery after the COVID-19 pandemic, as well as preparing for the unknown risks of the next wave of isolation regulations (43).

### Limitations

This study had several limitations. First, although we found a clear relationship between experiences of home environment conditions and environmental landscapes on child health, we could not determine a causal relationship between these variables due to the cross-sectional nature of the study design. Therefore, further research using longitudinal studies is needed. Second, this study relied on self-reported data, which may lead to reporting bias such as under or over-reporting actual health outcomes or recall bias. We recommend further studies in this field to overcome this limitation. Third, since participation in our survey was voluntary, there was the possibility of non-response bias in which participants who chose to participate in the study had different personal characteristics than those who did not select to participate. Fourth, mentally disabled children were identified based only on parents' reports. Finally, since the home environment conditions and environmental landscapes affect on child health are different among regions with different socioeconomic and cultural backgrounds (44), caution should be used to generalize the results to populations outside of Iran. Therefore, we recommend that similar studies be conducted in other regions worldwide.

## Conclusion

The results show that the home environment conditions and environmental landscapes affected child health, and the children of families whose exterior landscape of the home was nature had better health. Furthermore, the children's health of people whose home environment landscape had a garden, compared to the other two groups (yard, terrace), had better health. Home gardens are a potential source of health and are not necessarily replaced with other natural environments. In addition, providing them along with green space, is one of the crucial issues that should be considered. The results of our study can help health decision-makers in developing potential quarantine measures in the future to reduce the adverse effects of pandemics.

## Data availability statement

The original contributions presented in the study are included in the article/supplementary material, further inquiries can be directed to the corresponding author.

## Ethics statement

The studies involving human participants were reviewed and approved by the Ethics Committee at the University of Social Welfare and Rehabilitation Sciences in Tehran (IR.USWR.REC.1400.058). The patients/participants provided their written informed consent to participate in this study. Written informed consent was obtained from the individual(s) for the publication of any potentially identifiable images or data included in this article.

## Author contributions

MG and HS were responsible for the study conceptualization and led the paper's writing. GGAH, CC, AZ, and SA conducted the literature review and assisted in writing the paper. SAA, AZ, and SA performed the analysis, assisted in interpreting the data, and writing the paper. MG, PS, and GGAH assisted with the interpretation of the results and drafting programmatic implications. AZ, SAA, MA, and SA were responsible for the data collection and coordination of the study. MG co-led the conceptualization, supervised all aspects of writing the paper, and provided extensive comments on the manuscript. All authors were responsible for the study and have read and approved the final manuscript.
